# Development of an Inactivated Avian Influenza Virus Vaccine against Circulating H9N2 in Chickens and Ducks

**DOI:** 10.3390/vaccines11030596

**Published:** 2023-03-05

**Authors:** Yuzhuo Liu, Dongmin Zhao, Jingfeng Zhang, Xinmei Huang, Kaikai Han, Qingtao Liu, Jing Yang, Lijiao Zhang, Yin Li

**Affiliations:** 1 Institute of Veterinary Medicine, Jiangsu Academy of Agricultural Sciences, Nanjing 210014, China; 2 GuoTai (Taizhou) Center of Technology Innovation for Veterinary Biologicals, Taizhou 225300, China; 3 Key Laboratory of Veterinary Biological Engineering and Technology, Ministry of Agriculture, Nanjing 210014, China; 4 College of Veterinary Medicine, Nanjing Agricultural University, Nanjing 210095, China; 5 Institute of Life Sciences, Jiangsu University, Zhenjiang 212013, China

**Keywords:** avian influenza virus, H9N2, inactivated vaccine, protective efficiency, chicken, duck

## Abstract

Avian influenza virus (AIV) subtype H9N2 is the most widespread AIV in poultry worldwide, causing great economic losses in the global poultry industry. Chickens and ducks are the major hosts and play essential roles in the transmission and evolution of H9N2 AIV. Vaccines are considered an effective strategy for fighting H9N2 infection. However, due to the differences in immune responses to infection, vaccines against H9N2 AIV suitable for use in both chickens and ducks have not been well studied. This study developed an inactivated H9N2 vaccine based on a duck-origin H9N2 AIV and assessed its effectiveness in the laboratory. The results showed that the inactivated H9N2 vaccine elicited significant haemagglutination inhibition (HI) antibodies in both chickens and ducks. Virus challenge experiments revealed that immunization with this vaccine significantly blocked virus shedding after infection by both homogenous and heterologous H9N2 viruses. The vaccine was efficacious in chicken and duck flocks under normal field conditions. We also found that egg-yolk antibodies were produced by laying birds immunized with the inactivated vaccine, and high levels of maternal antibodies were detected in the serum of the offspring. Taken together, our study showed that this inactivated H9N2 vaccine could be extremely favourable for the prevention of H9N2 in both chickens and ducks.

## 1. Introduction

Avian influenza virus (AIV) belongs to the orthomyxoviridae family and influenza A virus genus. It is a single-stranded, negative-sense RNA virus consisting of eight gene fragments: PB1, PB2, PA, HA, NP, NA, M and NS. Different AIV strains can be classified into subtypes based on viral surface glycoproteins, haemagglutinin (HA) and neuraminidase (NA). There are currently 16 HA (H1 to H16) and 9 NA (N1 to N9) subtypes circulating in birds, and the subtypes H17N10 and H18N11 have been detected in bats [1,2,3]. Different AIV strains can cause from 10% to 100% mortality among poultry [4]. Based on their differences in virulence, AIVs can be divided into two main pathotypes, highly pathogenic avian influenza viruses (HPAIVs) and low-pathogenic avian influenza viruses (LPAIVs) [5]. Outbreaks of HPAIVs often result in severe systemic disease and up to 100% mortality in poultry, leading to great economic losses in poultry farming. Although HPAIVs evolve from LPAIVs of the H5 and H7 subtypes through genetic mutations, one LPAIV subtype, H9N2, should be given more attention [6,7].

Currently, H9N2 is the most widespread AIV subtype in poultry worldwide. In China, H9N2 was first isolated in chickens in 1994 and quickly spread to most areas of the country. Although H9N2 infection does not induce obvious clinical symptoms or cause high mortality, its prevalence in the poultry industry results in great economic losses due to reductions in egg production, decreases in body weight and coinfection with other pathogens [8]. H9N2 infection also induces transient immunosuppression, which may exacerbate other concomitant or secondary infections [9]. H9N2 has a wide host range, infecting birds, horses, pigs, dogs, ferrets, minks and humans [10]. In China, H9N2 has been detected in multiple avian species, including chickens, domestic waterfowl and pigeons [11]. Importantly, human H9N2 infections have been reported globally and have shown an increasing trend in recent years. Studies have demonstrated that all H9N2 viruses isolated from humans are of avian origin and genetically similar to those prevalent in local poultry at the same time [12]. Therefore, the threat of H9N2 to public health should be highlighted. In addition, owing to its broad host spectrum, reassortment between H9N2 and other influenza viruses is possible. Novel reassortant AIVs are frequently generated by acquiring internal gene segments from H9N2 viruses. For instance, the H7N9 virus, which infected humans in China in 2013, is presumed to have gained six internal genes from two different groups of H9N2 viruses [13]. The human and poultry H5N1 AIVs found in Hong Kong in 1997 were reassortant and obtained internal gene segments from the H9N2 virus (Qa/HK/G1/97) [14]. Such potential for reassortment remains a public health concern.

Wild aquatic birds are regarded as natural reservoirs and carriers of AIVs and play a critical role in the transmission and evolution of H9N2. Domestic ducks serve as intermediaries between wild aquatic birds and terrestrial poultry species [15]. Ducks infected with H9N2 develop only mild disease and are generally overlooked due to the lack of clinical symptoms. However, H9N2 can survive for long periods as a persistent infection and be shed into the environment through faeces [16]. Thus, effective control of H9N2 in ducks is important to prevent the prevalence of H9N2 in poultry.

Vaccination has been proven to be an effective way to protect poultry birds from AIV infection. Research and clinical data have shown that inactivated whole-virus H9N2 vaccines can provide protection for immunized chickens by alleviating clinical symptoms [11]. Nevertheless, antigenic drift frequently occurs on the H9N2 HA molecule. To provide enough protection, the H9N2 vaccine needs to be optimized to fit the strain currently circulating in chickens. In addition, vaccines against H9N2 in ducks are scarce. Compared with immunized chickens, the haemagglutination inhibition (HI) antibody titres induced in ducks by inactivated vaccines are lower and vary widely among individuals [17]. Moreover, vaccine-induced nonprotective antibodies cannot effectively eliminate the virus and block viral shedding. Therefore, an effective vaccine is urgently needed to improve serum antibody titres and prevent the spread of the H9N2 virus in ducks.

In the present study, an inactivated H9N2 vaccine was developed based on the H9N2 A/duck/Nanjing/01/1999 (NJ01) virus. The duck-origin H9N2 AIV was propagated in specific-pathogen-free (SPF) chicken embryos, inactivated with formaldehyde and emulsified with white oil adjuvant. Subsequently, the vaccine was assessed in chickens and ducks for its protective efficiency against H9N2 infection.

## 2. Materials and Methods

### 2.1. Viruses and Animals

Nine-day-old SPF chicken embryos and twenty-eight-day-old SPF chickens were purchased from the SPF Chicken Research Center of Shandong Institute of Poultry Science (Shandong, China). Twenty-eight-day-old SPF ducks were obtained from Harbin Veterinary Research Institute, China. This study was approved by the Animal Ethics Committee of Jiangsu Academy of Agricultural Sciences and followed the guidelines of animal experimentation outlined by Jiangsu Province (SYXK-Su-2020-0023). The H9N2 viruses A/Duck/Nanjing/01/1999 (NJ01), A/Duck/Anhui/01/2010 (AH01) and A/Chicken/HeNan/03/2009 (HN03) used in this study were isolated from clinically infected and dead ducklings or chickens in Jiangsu Province by our laboratory. The viruses were propagated in 9-day-old SPF chicken embryos. The 50% egg infectious dose (EID_50_) of each H9N2 virus (NJ01, AH01 and HN03) was calculated using Reed–Muench’s method [18]. The birds used in this study were confirmed to be free of any AIV infection, and different experimental groups were housed separately in SPF isolators.

### 2.2. Preparation of Vaccine Candidate

The master stock virus (10^8.5^ EID_50_/0.1 mL) was diluted 1:10,000 and inoculated into 9-day-old SPF chicken embryos. The allantoic fluid was harvested and inactivated with formaldehyde (final concentration of 0.2%) at 37 °C for 30 h. Complete inactivation of virus infectivity was confirmed by prolonged passages in 9-day-old SPF chicken embryos. Inactivated virus was suspended in Tween-80 with a volume ratio of virus to Tween-80 of 96:4. The oil phase consisted of medicinal-grade white oil and Span-80 at a volume ratio of 94:6 (white oil to Span-80). To obtain a water-in-oil emulsion vaccine, one volume of aqueous-phase was emulsified with three volumes of oil-phase adjuvant [19].

### 2.3. Antibody Responses after Immunization

Twenty-eight-day-old SPF chickens were randomly divided into two groups (ten per group): an immunized group and a negative control group. Each chicken in the immunized group was given 0.3 mL of inactivated vaccine subcutaneously in the neck. Ten twenty-eight-day-old SPF ducks were immunized with the same dose of inactivated vaccine in the same manner. The chickens and ducks that did not receive any treatment served as the negative control groups. At 3, 8, 12, 17, 21 and 25 weeks after immunization, sera were collected from all the birds and antibodies were detected by the haemagglutination inhibition (HI) test.

### 2.4. Protective Efficacy

Twenty-eight-day-old SPF chickens were randomly divided into six groups (ten per group) and immunized subcutaneously in the back of the neck. Group A1, Group A2, Group A3, Group A4 and Group A5 received 0.025 mL, 0.05 mL, 0.1 mL, 0.2 mL and 0.3 mL of inactivated vaccine, respectively. Twenty-eight-day-old SPF ducks were immunized with the same doses of inactivated vaccine in the same manner. Chickens and ducks given no treatment were used as negative controls. Three weeks after immunization, sera were collected from all birds for antibody detection using the HI test, and then all chickens and ducks were mixed and reorganized into groups based on the HI titre. Each group of birds was then experimentally challenged with 10^8.0^ EID_50_ NJ01 virus by intravenous injection. Oropharyngeal swabs were collected from each duck on days 1 and 2 postchallenge, and cloacal swabs were collected from each chicken on day 5 postchallenge. The oropharyngeal and cloacal swab samples were inoculated into 9-day-old SPF chicken embryos for virus isolation.

### 2.5. Determination of Minimum Protective Dose

Twenty-eight-day-old SPF chickens were randomly assigned to six groups of ten birds each. The chickens in Groups B1 to B5 were vaccinated with 0.025 mL, 0.05 mL, 0.1 mL, 0.2 mL and 0.3 mL of inactivated vaccine, respectively. The chickens in Group B6 served as controls. Twenty-eight-day-old SPF ducks were vaccinated with the same doses of inactivated vaccine in the same manner. Three weeks after vaccination, serum samples were collected from all birds for antibody detection.

### 2.6. Cross-Protective Efficacy of Inactivated Vaccine against H9N2 Viruses

For cross-protection experiments, twenty-eight-day-old SPF chickens were randomly divided into four groups with ten chickens in each group. The chickens in Groups C1 and C2 were vaccinated with 0.3 mL of the inactivated vaccine. The chickens in Groups C3 and C4 served as controls. In week 3 postvaccination, Groups C1 and C3 were challenged with H9N2 AIV strain AH01 intravenously at a dose of 10^8.0^ EID_50_ per bird, and Groups C2 and C4 were challenged with H9N2 AIV strain HN03 intravenously. Twenty-eight-day-old SPF ducks were vaccinated and challenged in the same manner. Samples were obtained for virus isolation as described in Section 2.4.

### 2.7. Field Application

The vaccine was tested on three chicken farms and three duck farms. A total of 3 flocks with a minimum of 18,000 chickens or ducks between 10 and 70 days of age were used in the trial, and the serological response was monitored in week 3 postvaccination. Analyses were carried out on 1% of the vaccinated population.

### 2.8. Detection of Egg Yolk Antibody and Maternal Antibody

Laying hens or ducks (120 days old) were immunized with 0.5 mL of inactivated vaccine. Sera and eggs were collected for antibody evaluation in week 3 postimmunization.

For assessment of maternal antibody transfer, 200-day-old breeding hens or ducks were vaccinated with 0.8 mL of inactivated vaccine. At 3 weeks postvaccination, eggs were collected and incubated for 21 (chicken eggs) or 28 (duck eggs) days. After hatching, the chicks and ducklings were bled for sera collection on days 1, 3, 6, 9 and 12.

### 2.9. Statistical Analysis

Data were statistically analysed using Statistics Package for Social Science Statistical (SPSS). Data are expressed as the mean ± SD of ten birds.

## 3. Results

### 3.1. Antibody Responses after Immunization

**Figure 1:**
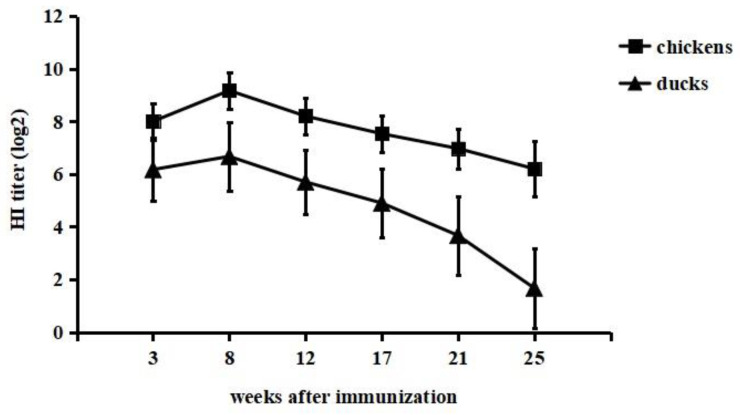
Antibody responses in chickens and ducks immunized with inactivated vaccine. Twenty-eight-day-old SPF chickens and ducks were given the developed inactivated vaccine subcutaneously. At 3, 8, 12, 17, 21 and 25 weeks after immunization, sera were collected for HI antibody determination by the haemagglutination inhibition (HI) test.

Serum antibody responses to immunization were detected by the HI test. Chickens and ducks in the immunized groups showed rapid increases in HI titres following vaccine immunization (Figure 1). The HI titre in chickens of the immunized group was higher than that in immunized ducks. The peak HI titre in chickens was 9.2 log2 at 8 weeks after immunization, while the HI titre in ducks reached a maximum of 6.7 log2 at the same time-point. The HI titres in chickens were still higher than 6 log2 at 25 weeks postimmunization. However, ducks showed titres of more than 6 log2 until 12 weeks postimmunization, and afterwards, there was a decrease in the titre level. The chickens and ducks in the control groups did not elicit HI antibodies. From these results, we concluded that the inactivated vaccine elicited high titres of H9-specific HI antibodies in both chickens and ducks, and immunized chickens exhibited a higher antibody peak level and longer duration of antibody presence than immunized ducks. In addition, no swelling or induration was observed at the inoculation site throughout the experimental period, suggesting that this vaccine was effective and safe.

### 3.2. Protective Efficacy after Viral Challenge

**Figure 2:**
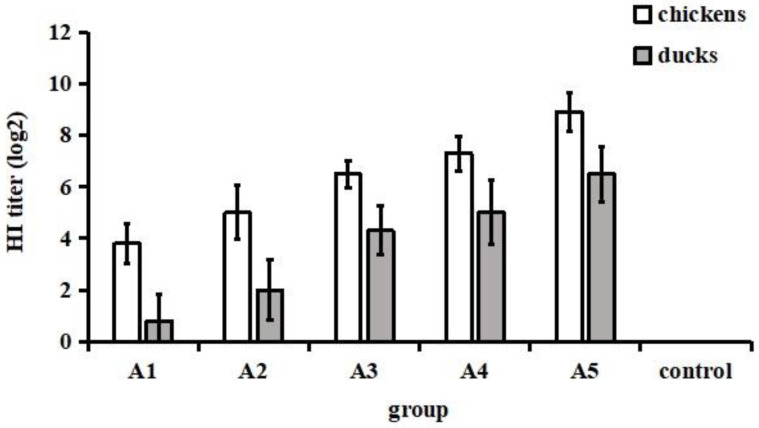
HI antibody titres in chickens and ducks immunized with different volumes of inactivated vaccine. Twenty-eight-day-old SPF chickens and ducks were given the developed inactivated vaccine subcutaneously. At three weeks after immunization, sera were collected for HI antibody determination by the haemagglutination inhibition (HI) test.

The chickens that received various volumes of vaccine produced different levels of HI antibodies ranging from 3 log2 to 10 log2. Moreover, the vaccine-induced HI antibody titres in duck sera were between 0 log2 and 8 log2 (Figure 2). Based on the HI titre, chickens and ducks were regrouped into eight groups and nine groups, respectively, and intravenously challenged with the NJ01 strain.

After challenge, the positive rate of virus isolation reached 100% (10/10) in the control group of unimmunized chickens (Table 1). In the group with HI titres of 3, 4 and 5 log2, the positive rate of virus isolation ranged from 80% (4/5) to 16.7% (1/6). In contrast, no viruses were isolated from SPF chicken embryos inoculated with cloacal swab samples from chickens in the group with HI titres greater than 6 log2, resulting in a protection rate of 100%. The results indicated that the lowest HI titre for chickens against H9N2 infection was 6 log2. The correlation between the HI titre and efficacy in ducks was slightly different from that in chickens. The minimum HI titre required to block viral shedding was 4 log2 (Table 2).

### 3.3. Determination of Minimum Protective Dose

The minimum protective doses of the vaccine were determined in chickens and ducks. Twenty-eight-day-old SPF chickens and ducks were subcutaneously inoculated with 0.025 mL, 0.05 mL, 0.1 mL, 0.2 mL and 0.3 mL of inactivated vaccine (equivalent to 10^7.1^, 10^7.4^, 10^7.7^, 10^8.0^ and 10^8.2^ EID_50_, respectively). At 3 weeks after vaccination, birds in all groups were bled for detection of HI antibodies. According to the correlation between HI titre and efficacy, the chickens and ducks vaccinated with 10^7.4^ EID_50_ were only partially protected (40% and 10%, respectively), whereas chickens vaccinated with 10^7.7^ EID_50_ showed complete protection (Table 3). Vaccination with 10^7.7^ and 10^8.0^ EID_50_ provided good protection (80%-90%) for ducks and vaccination with 10^8.2^ EID_50_ gave full protection. Accordingly, for both chickens and ducks, the minimum protective dose of inactivated vaccine was 10^7.7^ EID_50_. To assure consistent, strong protection under various conditions, it is suggested that the dose of NJ01 used to vaccinate chickens and ducks should not be less than 10^8.2^ EID_50_.

### 3.4. Cross-Protective Efficacy of Inactivated Vaccine against Heterologous H9N2 Viruses

To evaluate whether the inactivated vaccine could also elicit cross-protection against challenge with heterologous viruses, each vaccinated group of chickens and ducks was challenged with 10^8.0^ EID_50_ AH01 or HN03 intravenously. During the chicken studies, all ten immunized chickens were protected against AH01 and HN03 challenge when compared to control unimmunized chickens, as evidenced by the fact that no virus was isolated from the cloacal swabs collected from the immunized birds, while the virus was detected in all cloacal swabs collected from the unimmunized chickens. Similarly, all ducks from the immunized groups were effectively protected against AH01 and HN03 challenge. Conversely, ducks in the control group showed white- or green-coloured diarrhoea 1-day postchallenge, and virus was recovered in all oropharyngeal swabs collected from the unimmunized ducks (Table 4). These data showed that the inactivated vaccine can provide cross-protection against heterologous H9N2 virus infection in both chickens and ducks.

### 3.5. Field Application

The inactivated vaccine was tested on a large scale under normal field conditions in three flocks of chickens and ducks. The results are presented as the percentage of seropositive birds as tested by the HI assay (Table 5). The global serological response obtained by the HI test on three chicken farms reached 100% at 3 weeks after vaccination. The percentage of seropositive ducks was higher than 90%, with some differences between the three farms. These results suggest that this inactivated vaccine could provide adequate protection against H9N2 AIV for chickens and ducks in the field.

### 3.6. Determination of Egg Yolk Antibody and Maternal Antibody

As shown in Table 6, all serum and yolk samples were positive for HI antibodies to H9N2 3 weeks after immunization in the vaccinated groups. Samples from unimmunized birds remained negative. The serum antibody titre was slightly higher than the yolk antibody titre, and both were positively correlated. Table 7 presents the results of maternal antibody determination by the HI test. At hatching, both chicks and ducklings had significant levels of antibody in serum and reached HI antibody titres of 8.6 and 7.3 log2, respectively. The titres of maternal antibodies in the serum declined gradually over the course of the experiment. The data showed that maternally derived antibodies provided good protection to the chicks and ducklings during the first 9 and 6 days of life, respectively.

## 4. Discussion

It is commonly accepted that the wide spread of H9N2 AIV is becoming a public health concern because of the extension of its host spectrum, virulence enhancement and ability to provide internal genes for reassortment with AIVs of other subtypes [20]. Evidence has shown that H9N2 AIV gradually suppresses other subtypes and becomes dominant in both chickens and ducks [21]. In China, H9N2 has established stable lineages in commercial chicken flocks [22]. As a natural reservoir of H9N2, ducks play an important role in contributing to the spread and evolution of H9N2 AIV. Additionally, ducks may act as incubators for creating novel reassortants [23]. Although an inactivated vaccine against H9N2 AIV has been continuously used since 1998, circulating H9N2 strains pose a challenge to the effectiveness of this vaccine. In addition, current commercial vaccines against H9N2 AIV cannot provide satisfactory antibody titres and protective immunity in ducks [24]. Thus, more effective vaccines are urgently needed to prevent and control the prevalence of H9N2 in both chickens and ducks.

To date, all of the vaccine strains used in commercial H9N2 AIV vaccines have been isolated from chickens [11]. Here, a new inactivated vaccine was developed based on a duck-origin H9N2 AIV strain (NJ01), and its protective efficacy was evaluated in both chickens and ducks. The immunogenicity of the inactivated vaccine was assessed based on HI antibody titres. The HI antibody titre of all chickens and ducks vaccinated with inactivated vaccine increased rapidly, reached their highest levels after eight weeks and then began to decline, but the level was still above the protective threshold until at least 17 weeks after vaccination. A previous study showed that HI antibodies against H9N2 AIV elicited by an inactivated bivalent vaccine was no longer able to provide protection to chickens 15 weeks postvaccination [19]. In contrast, Teng et al. reported that vaccinated ducks maintained high antibody levels from 1-week postvaccination to 37 weeks postvaccination and provided complete protection from oropharyngeal viral shedding after challenge with H9N2 AIV [25]. The reason for these results may be that the seed virus was inactivated by beta-propiolactone (BPL) and mixed with the adjuvant Montanide ISA 70VG. BPL has been demonstrated to have a significantly higher degree of antigenicity and to be more protective in inactivated vaccines than other chemicals [26]. The adjuvant Montanide ISA 70VG can induce better immune effects in chickens and ducks [27]. There are many conventional and novel methods for inactivating influenza viruses. One of the chemicals that is frequently used to produce whole inactivated vaccines is formaldehyde. Another inactivating compound that is used by many vaccine producers is BPL. Unlike formaldehyde, BPL is able to create bis-alkylated complexes in high yields through the introduction of inter- and intraprotein crosslinking. In this way, the influenza virus HA and NA enzymatic activities may be greatly decreased, if not extinguished completely [28]. Thus, it is worth investigating whether BPL and 70VG enhance the immunogenicity of our vaccine.

Vaccination is the main strategy used to control the spread of H9N2 AIV in chicken and duck flocks. The data show that all certified H9-vaccine-related products were inactivated whole-virus vaccines [11]. However, multiple H9N2 viruses with different antigenicities are prevalent in China, making current vaccination against H9N2 challenging. Given that vaccine candidate strains are critical for inducing vaccine immunity, viruses with cross-immune protection characteristics should be used to prepare inactivated vaccines. Ducks and chickens are AIV hosts, and each has a distinctive response to H9N2 infection [29]. Ducks normally produce lower antibody responses to vaccination with AIV vaccines due to the unusual features of duck antibodies [29]. In this study, the inactivated vaccine elicited higher HI antibody titres in chickens than in ducks, which is in agreement with earlier studies [30]. It is generally accepted that ducks need a larger vaccine dose than chickens. Our results demonstrated that chickens and ducks that received the same dose of vaccine can resist infection by duck- and chicken-origin H9N2 viruses, suggesting that this vaccine is more favourable for the control of H9N2 AIV in both chickens and ducks.

Considering that animal conditions vary greatly depending on the individual farm, the efficacy of vaccines should be tested in the field prior to their introduction into the market. We evaluated the global serological responses to this inactivated vaccine in chicken and duck flocks from different farms. After large-scale vaccination under normal field conditions, the serology data suggested that positive immune responses were stimulated in vaccinated birds, and the HI antibody titres were similar to those on the laboratory scale. This vaccine could be an efficient tool contributing to simplifying AIV vaccination in poultry.

Female birds can transfer AIV-specific antibodies to their offspring via the egg yolk [31]. The level of AIV antibodies in egg yolk reflects the level of circulating AIV antibodies in the mother at the time of transfer, which was confirmed by our research. Since animal welfare is an issue of great concern, there is a need for alternative sources of antibodies that could be produced without causing stress to animals. Egg yolk is a good alternative to serum when detecting AIV antibodies. In addition, a previous study demonstrated that passive administration of egg-yolk antibodies can reduce the morbidity and virus shedding of H9N2 AIV [32]. Antigens are one of the most important factors in the development and production of specific egg-yolk antibodies [33]. Our results showed that the inactivated vaccine induced a high titre of egg-yolk antibodies, suggesting that this vaccine has wide application value in preparing egg-yolk antibodies.

Egg-yolk antibodies are absorbed and enter the circulatory system of offspring and are known as maternal antibodies. Maternal antibodies can protect young birds from pathogen infection. In this study, the antibody titres in chicks and ducklings derived from immunized breeding birds remained above the protection threshold value at 9 and 6 days of age, respectively. Moreover, maternal antibodies are known to interfere with antigen processing, which reduces vaccination efficacy in poultry [34]. To overcome this interference, our research recommends vaccinating chicks between 9 and 12 days of age, and vaccination of ducklings should start at 6 to 9 days of age. Further studies are required to optimize the vaccination schedule for field application.

## 5. Conclusions

This study developed an inactivated vaccine that provides complete protection from H9N2 AIV infection in both chickens and ducks. The vaccine elicited a significant and prolonged antibody response in vaccinated chickens and ducks and good protection efficacy against the circulating strain of H9N2 AIV. The egg-yolk and maternal antibodies were produced by immunizing breeding birds with the inactivated vaccine. Therefore, this vaccine could be a potential vaccine for application in the poultry industry to control the prevalence of H9N2 AIV.

## Figures and Tables

**Table 1:** Protective efficacy against H9N2 challenge in chickens.

HI Antibody Titres (log2)	Virus Isolation/Total	Protection Rate (%)
3	4/5	20
4	4/6	33
5	1/6	83
6	0/10	100
7	0/10	100
8	0/8	100
9	0/5	100
10	0/2	100
Control	10/10	0

**Table 2:** Protective efficacy against H9N2 challenge in ducks.

HI Antibody Titres (log2)	Virus Isolation/Total	Protection Rate (%)
0	4/6	33
1	2/5	60
2	1/5	80
3	0/6	100
4	1/8	87.5
5	0/7	100
6	0/7	100
7	0/4	100
8	0/2	100
Control	8/10	20

**Table 3:** The HI antibody titres of chickens and ducks immunized with various doses of inactivated vaccine.

Group	Vaccination Volume (mL)	Dose (EID_50_)	Chickens	Ducks
			The Ratio of HI TITRE ≥6 log2 (%)	The Ratio of HI Titre ≥4 log2 (%)
B1 B2	0.025 0.05	10^7.1^	0/10 (0)	0/10 (0)
		10^7.4^	4/10 (40%)	1/10 (10%)
B3	0.1	10^7.7^	10/10 (100%)	8/10 (80%)
B4	0.2	10^8.0^	10/10 (100%)	9/10 (90%)
B5	0.3	10^8.2^	10/10 (100%)	10/10 (100%)
Control	-	-	0/10 (0)	0/10 (0)

EID_50_: 50% egg infectious dose; HI: haemagglutination inhibition.

**Table 4:** Evaluation of cross-protective efficacy of vaccine against H9N2 viruses.

Group	Immunization	Field Strain	Chickens		Ducks	
			Mean HI Titre (log2)	Virus Isolation /Total (%)	Mean HI Titre (log2)	Virus Isolation /Total (%)
C1 C2	Vaccine Vaccine	AH01	8.9 ± 0.74	0/10 (0)	5.1 ± 0.94	0/10 (0)
		HN03	9.0 ± 0.67	0/10 (0)	4.9 ± 0.83	1/10 (10%)
C3	-	AH01	0	10/10 (100%)	0	10/10 (100%)
C4	-	HN03	0	10/10 (100%)	0	10/10 (100%)

HI: haemagglutination inhibition.

**Table 5:** Percentage of seroconversion of vaccinated birds by the inactivated vaccine in the farms.

Farm	Immunization	Chickens		Ducks	
		The Ratio of HI Titre ≥ 6 log2 (%)	Mean HI Titre (log2)	The Ratio of HI Titre ≥ 4 log2 (%)	Mean HI Titre (log2)
No. 1	Vaccine	100	7.9 ± 0.54	100	5.1 ± 1.01
	-	0	0	0	0
No. 2	Vaccine	100	8.5 ± 0.86	90	4.3 ± 1.44
	-	0	0	0	0
No. 3	Vaccine	100	7.8 ± 0.97	100	5.6 ± 1.05
	-	0	0	0	0

HI: haemagglutination inhibition.

**Table 6:** The titres of egg yolk antibody at 3 weeks after immunization.

	Immunization		Serum	Yolk
Chickens	Vaccine	The ratio of HI titre ≥ 6 log2 (%)	100	100
		Mean HI titre (log2)	9.75 ± 0.71	9.2 ± 0.75
	-	The ratio of HI titre ≥ 6 log2 (%)	0	0
		Mean HI titre (log2)	0	0
Ducks	Vaccine	The ratio of HI titre ≥ 4 log2 (%)	100	100
		Mean HI titre (log2)	8.7 ± 1.02	8.4 ± 0.99
	-	The ratio of HI titre ≥ 4 log2 (%)	0	0
		Mean HI titre (log2)	0	0

HI: haemagglutination inhibition.

**Table 7:** The dynamics of maternal antibody in the offspring.

Age (Day)	Immunization	Chicks		Ducklings	
		The Ratio of HI Titre ≥ 6 log2 (%)	Mean HI Titre (log2)	The Ratio of HI Titre ≥ 4 log2 (%)	Mean HI Titre (log2)
1	Vaccine	100	8.6 ± 0.87	100	7.3 ± 0.82
	-	0	0	0	0
3	Vaccine	100	7.6 ± 0.82	100	5.9 ± 0.85
	-	0	0	0	0
6	Vaccine	90	6.9 ± 0.71	80	4.4 ± 0.97
	-	0	0	0	0
9	Vaccine	90	6.1 ± 0.60	50	3.1 ± 0.82
	-	0	0	0	0
12	Vaccine	40	5.3 ± 0.67	10	1.1 ± 0.74
	-	0	0	0	0

HI: haemagglutination inhibition.

## Data Availability

All the data generated or analysed in this study can be obtained by contacting the corresponding author.
